# Hypothalamic gene transfer of BDNF promotes healthy aging in mice

**DOI:** 10.1111/acel.12846

**Published:** 2018-12-26

**Authors:** Travis McMurphy, Wei Huang, Xianglan Liu, Jason J. Siu, Nicholas J. Queen, Run Xiao, Lei Cao

**Affiliations:** ^1^ Department of Cancer Biology and Genetics, College of Medicine The Ohio State University Columbus Ohio; ^2^ The Ohio State University Comprehensive Cancer Center Columbus Ohio

**Keywords:** adipose tissue, aging, BDNF, gene transfer, hypothalamus, steatosis

## Abstract

The aging process and age‐related diseases all involve perturbed energy adaption and impaired ability to cope with adversity. Brain‐derived neurotrophic factor (BDNF) in the hypothalamus plays important role in regulation of energy balance. Our previous studies show that recombinant adeno‐associated virus (AAV)‐mediated hypothalamic BDNF gene transfer alleviates obesity, diabetes, and metabolic syndromes in both diet‐induced and genetic models. Here we examined the efficacy and safety of a built‐in autoregulatory system to control transgene BDNF expression mimicking the body's natural feedback systems in middle‐aged mice. Twelve‐month‐old mice were treated with either autoregulatory BDNF vector or yellow fluorescence protein (YFP) control, maintained on normal diet, and monitored for 28 weeks. BDNF gene transfer prevented the development of aging‐associated metabolic declines characterized by: preventing aging‐associated weight gain, reducing adiposity, reversing the decline of brown fat activity, increasing adiponectin while reducing leptin and insulin in circulation, improving glucose tolerance, increasing energy expenditure, alleviating hepatic steatosis, and suppressing inflammatory genes in the hypothalamus and adipose tissues. Moreover, BDNF treatment reduced anxiety‐like and depression‐like behaviors. These safety and efficacy data provide evidence that hypothalamic BDNF is a target for promoting healthy aging.

## 1 INTRODUCTION

The natural aging process and many age‐related diseases are associated with impaired metabolic adaption and declined ability to cope with stress. The evolution theory of aging states that mechanisms that decrease the probability of dying from environmental hazards and disease would be expected to lead to increased longevity (Robins & Conneely, 2014). From this perspective, the brain plays a commanding role. Accumulating evidence suggests that there may be a discrete number of conserved neuronal signaling pathways that determine healthspan via regulating energy metabolism and resistance to distress. Mattson and colleagues propose that a neural signaling triumvirate: insulin/IGF‐1, BDNF, and serotonin may be important determinants of health during aging because of their cooperative influence on energy metabolism, stress response, and cardiovascular function (Mattson, Maudsley, & Martin, 2004). BDNF has diverse functions in brain development and plasticity (Lu, Pang, & Woo, 2005). It is neuroprotective in many different brain areas against dysfunctions and insults (Lindsay, 1994). In addition, BDNF is an important component of the hypothalamic pathway that controls energy homeostasis (Xu & Xie, 2016). We have previously demonstrated that an enriched environment (EE) improves brain function and the body's overall state of health. Our mechanistic studies lead to the characterization of a novel brain‐fat axis—the hypothalamic‐sympathoneural‐adipocyte (HSA) axis, and the development of molecular therapy for obesity, diabetes, and cancer. The complex stimuli (physical, social, and cognitive) provided by EE induce hypothalamic BDNF and elevate sympathetic tone to the adipose tissue. Increased sympathetic tone remodels the adipose tissue, inducing browning of white fat and suppression of leptin, leading to an anti‐obesity and anticancer phenotype (Cao & During, 2012; Cao et al., 2011, 2009, 2010 ). Furthermore, hypothalamic BDNF modulates secondary lymphoid tissues (spleen and lymph nodes) and enhances CD8 T cell immunity, contributing to the anticancer effects of EE (Xiao et al., 2016). A reduction in BDNF signaling has been documented during normal aging and decreased BDNF levels are associated with vulnerable neuronal populations in several neurodegenerative disorders including Alzheimer's, Parkinson's and Huntington's diseases, demonstrating the need for further therapeutic research on components of the BDNF signaling pathway (Tapia‐Arancibia, Aliaga, Silhol, & Arancibia, 2008). Some physiologic or pathologic age‐related changes in the CNS could be offset by the administration of exogenous BDNF and/or by stimulation of its receptor expression (Tapia‐Arancibia et al., 2008). In addition, BDNF signaling in the brain is thought to mediate at least some of the anti‐aging effects of an intermittent fasting regiment (Duan et al., 2003; Lee, Duan, & Mattson, 2002) although data on the hypothalamus are not reported. Moreover, it is unclear how BDNF signaling in neurons is transferred to the periphery to improve the healthspan of many different organ systems. Our characterization of the HSA axis and the critical role of BDNF in this brain‐fat axis suggest a mechanism whereby hypothalamic BDNF, highly responsive to environmental stimuli, controls the HSA axis activity and thereby influences body composition, metabolism, immune function, and cancer via its preferential regulation of the phenotype and functions of adipose tissue. Here we investigated the long‐term effects of hypothalamic gene transfer of BDNF in middle‐aged mice using an autoregulatory rAAV vector. The single rAAV vector harbors two cassettes, one expresses human BDNF driven by a constitutive promoter, the other expresses a microRNA targeting BDNF under the control of agouti‐related peptide (AGRP) promoter that is activated by weight loss and fat depletion. This dual‐cassette vector mimics the body's natural feedback system to achieve autoregulation of the transgene and its efficacy has been examined in genetic models of obesity and diabetes such as *db*/*db* mice (Cao et al., 2009) and melanocortin‐4 receptor (MC4R) deficient mice (Siu et al., 2017).

## 2 RESULTS

### 2.1 Short‐term hypothalamic gene transfer of autoregulatory BDNF vector

**Figure 1:**
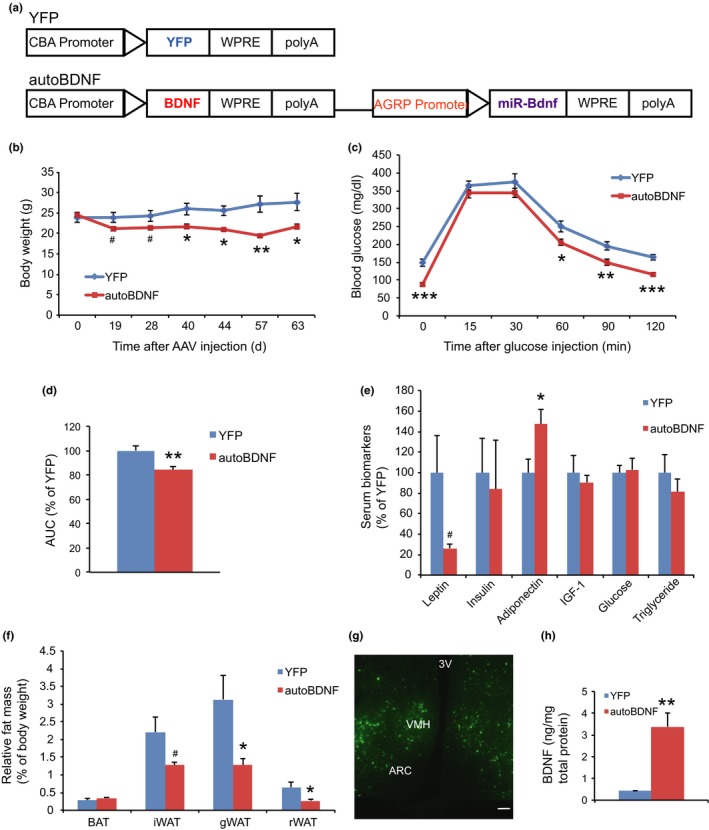
Vector construction and short‐term hypothalamic gene delivery. (a) AAV vector constructs used in study. CBA: cytomegalovirus enhancer plus chicken ß‐actin promoter; polyA: bovine growth hormone polyadenosine tail; WPRE: woodchuck posttranscriptional regulatory element. (b) Body weight. (c) Glucose tolerance test performed 57 days post‐AAV injection. (d) Area under the curve (AUC) of (c). (e) Relative fat mass at sacrifice 63 days post‐AAV injection. BAT: brown adipose tissue; gWAT: gonadal white adipose tissue; iWAT: inguinal white adipose tissue; rWAT: retroperitoneal white adipose tissue. (f) Serum biomarkers at sacrifice. (g) YFP fluorescence. Scale bar, 100 µm. ARC: arcuate nucleus; VMH: ventromedial hypothalamus; 3V: third ventricle. (h) BDNF protein level in hypothalamic dissections. Error bars represent mean ± *SEM*. *n* = 10 per group (b–e); *n* = 7 per group (h). *p < 0.05. **p < 0.01. ***p < 0.001. ^#^ *p < *0.06

To test the efficacy of hypothalamic BDNF gene transfer in middle‐aged mice, we performed a short‐term study. The autoregulatory dual‐cassette construct expressing the human BDNF (autoBDNF) or destabilized yellow fluorescent protein (YFP) control were packaged into serotype 1 AAV capsids (Cao et al., 2009; Figure 1a). Ten‐month‐old female C57BL/6 mice were randomized into the two treatment groups, to receive bilateral hypothalamic injections of either AAV‐ autoBDNF or AAV‐YFP into the arcuate (ARC)/ventralmedial (VMH) nuclei of the hypothalamus (Figure 1f). BDNF‐treated mice showed lower body weight compared to YFP mice (Figure 1b). A glucose tolerance test was performed 57 days post‐AAV injection and BDNF‐treated mice displayed improved glycemic control (Figure 1c,d). Mice were sacrificed 63 days post‐AAV injection and the intrascapular brown adipose tissue (BAT) and various white adipose tissue (WAT) including inguinal (iWAT), gonadal (gWAT), and retroperitoneal (rWAT) depots were dissected. BDNF treatment reduced adiposity with intra‐abdominal fat depots displaying the most reduction (Figure 1e). Profiling of serum biomarkers at the sacrifice showed a significantly higher adiponectin level and a strong trend toward lower leptin in BDNF mice (Figure 1f). YFP fluorescence confirmed that transgene expression was mainly in ARC/VMH nuclei (Figure 1g). The BDNF protein levels in the hypothalamus block dissections were measured using ELISA. The autoBDNF mice showed eightfold higher hypothalamic BDNF level than YFP mice (Figure 1h).

### 2.2 Systemic metabolic effects of long‐term hypothalamic gene transfer of BDNF

**Figure 2:**
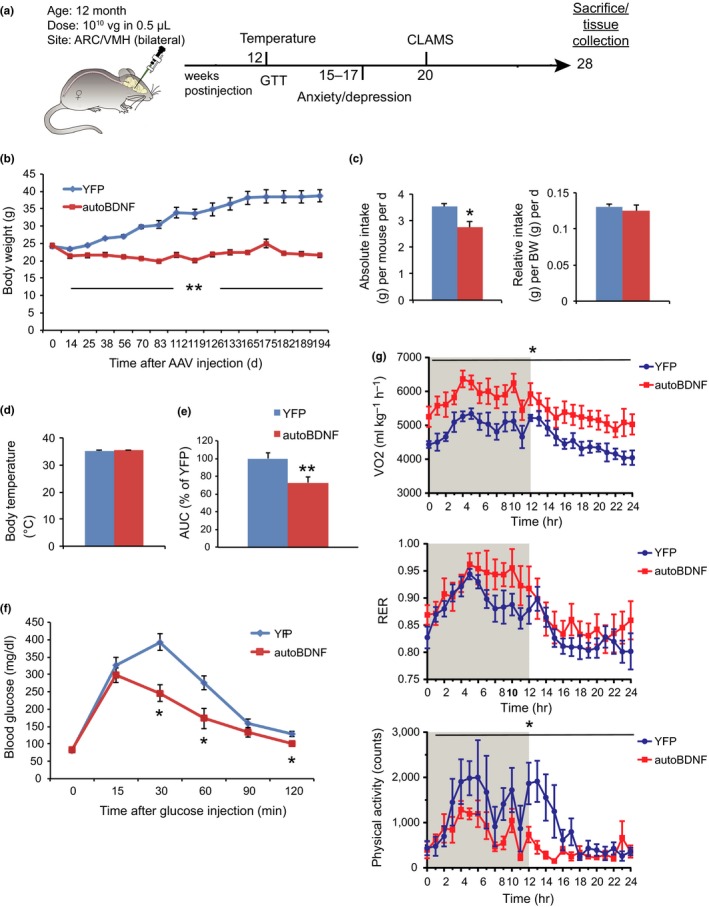
Metabolic effects of long‐term hypothalamic *BDNF *gene transfer. (a) Experimental design. (b) Body weight. (c) Absolute (left) and relative (right) food intake recorded from week 3 to 10 post‐AAV injection. (d) Core rectal temperature measured 12 weeks post injection. (e) Area under the curve (AUC) of (f). (f) Glucose tolerance test at 13 weeks post injection. *n* = 8–9 for YFP, *n* = 9–10 for autoBDNF (b) to (f). (g) CLAMS assessment at 20 weeks post injection. Oxygen consumption, respiratory exchange ratio (RER), and physical activity in a 24‐hr period; Shaded area, dark phase. *n* = 6 per group. Error bars represent mean ± *SEM*. *p < 0.05. **p < 0.01

Next, we conducted a long‐term study to assess the effects of hypothalamic BDNF overexpression on normal aging and more comprehensively characterize the metabolic and behavioral implications (Figure 2a). Twelve‐month‐old female C57BL/6 mice were randomized to receive AAV‐autoBDNF or YFP and monitored for 7 months. YFP‐treated mice gradually gained weight. In contrast, BDNF treatment completely prevented aging‐related weight gain (Figure 2b). Moreover, autoBDNF‐treated mice maintained stable body weight throughout the 7‐month duration of the study (Figure 2b). Food intake was monitored between week 3 and 10 postsurgery. The absolute food intake of BDNF‐treated mice was lower than YFP mice while the relative consumption calibrated to body weight was not different (Figure 2c). Rectal temperature measured at 12‐weeks post injection revealed no significant differences between the two groups (Figure 2d). At 13‐weeks postsurgery, BDNF‐treated mice performed better in a glucose tolerance test (Figure 2e,f).

To assess energy expenditure, mice were subjected to indirect calorimetry beginning 20 weeks post‐AAV injection over a 24‐hr period after habituation. Oxygen consumption in BDNF‐treated mice was significantly increased in both dark and light phases compared to YFP mice (Figure 2g). The respiratory exchange ratio (RER) was slightly increased in the BDNF group during the dark phase but was not statistically significant. (Figure 2g). Surprisingly, physical activity was significantly decreased in BDNF‐treated mice (Figure 2g). Food intake was not different during this period in the metabolic chambers (data not shown). The higher oxygen consumption concurrent with lower physical activity indicated that BDNF treatment elevated the resting metabolic rate.

### 2.3 Behavioral assessments of long‐term BDNF gene transfer

**Figure 3:**
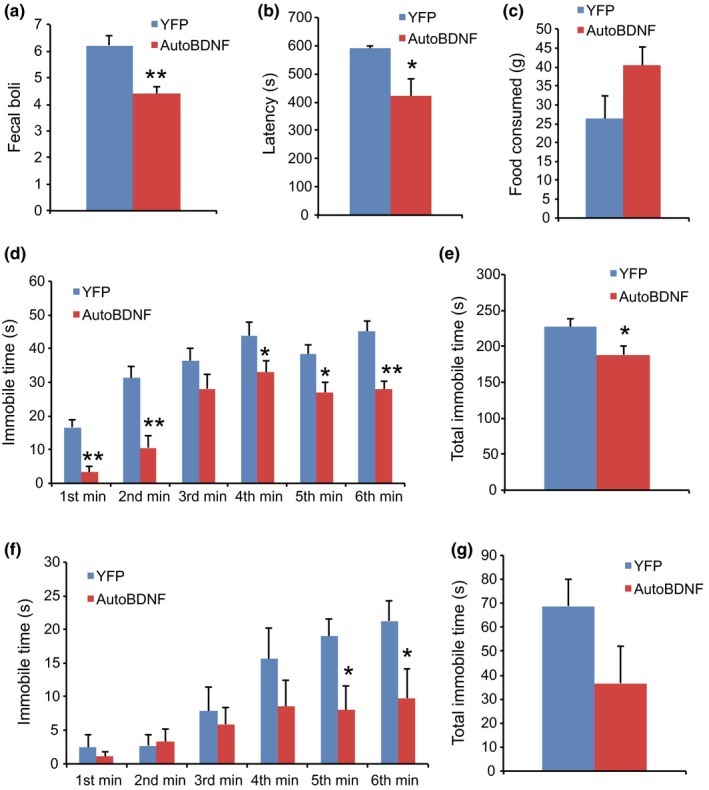
Behavioral effects of hypothalamic gene transfer of BDNF. (a) Number of fecal boli in the cold‐induced defecation test for anxiety. (b) Latency to feed in novelty‐suppressed feeding. (c) Amount of food consumed in novelty‐suppressed feeding. (d) Time spent immobile per minute of the tail suspension test. (e) Total immobility time during the tail suspension test. (f) Time spent immobile per minute of the forced swim test. (g) Total immobility time during the forced swim test. *n* = 9 for YFP, *n* = 10 for autoBDNF. Error bars represent mean ± *SEM*. *p < 0.05. **p < 0.01

In addition to assessing metabolic efficacy, we were interested in the long‐term safety of this approach in aged mice on normal diet. Thus, we performed various assays to screen for changes in anxiety‐ or depression‐like behavior from 15–17 weeks post‐AAV injection. In an anxiety behavior test, cold‐induced defecation (CID; Barone et al., 2008), BDNF mice showed significant reduction in fecal boli compared to YFP mice (Figure 3a). The novelty‐suppressed feeding (NSF) test assesses hyponeophagia, in which exposure to a novel environment suppresses feeding behavior (Samuels & Hen, 2011). NSF has been used to study anxiety‐ and depression‐related behaviors because it is sensitive to anxiolytic and chronic antidepressant treatments. BDNF treatment shortened the latency to feed (Figure 3b). Two assays for depression were used, the tail suspension (TST) and forced swim tests (FSTs). For the TST test, the time being immobile was significantly diminished in 5 min of the 6‐min test for BDNF‐treated mice (Figure 3d). The total amount of immobile time was also significantly reduced in the BDNF mice (Figure 3e). The FST is one of the most commonly used rodent behavioral tests for screening antidepressant drugs (Cryan & Mombereau, 2004). The time being immobile was significantly decreased in the last 2 min of the 6‐min test for BDNF‐treated mice (Figure 3f). The total immobile time of the FST revealed a trend toward lower immobility in BDNF mice (Figure 3g).

### 2.4 BDNF treatment promotes a lean phenotype

**Figure 4:**
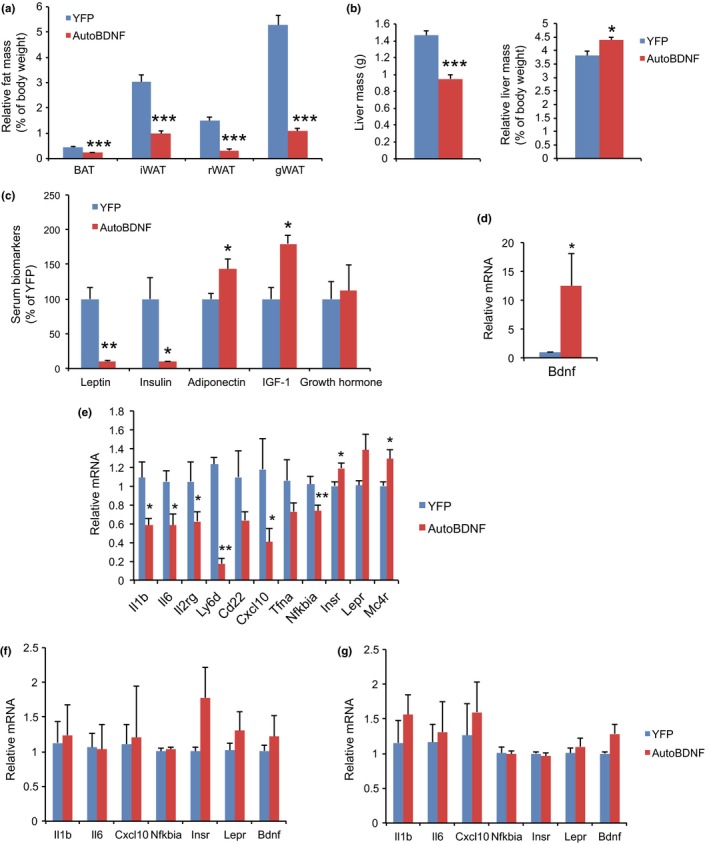
Tissue mass, serum biomarkers, and brain gene expression. (a) Relative fat mass at sacrifice 194 days post‐AAV injection. (b) Absolute (left) and relative (right) liver mass. (c) Serum biomarkers at sacrifice. (d) Hypothalamic BDNF expression. (e) Gene expression profiling of hypothalamus. (f) Gene expression profiling of amygdala. (g) Gene expression profiling of hippocampus. *n* = 8 for YFP, 9 for autoBDNF (a) to (c). *n* = 5 per group (d) to (g). Error bars represent mean ± *SEM*. *p < 0.05. **p < 0.01. ***p < 0.001

Mice were sacrificed 194 days post‐AAV injection. Significant reductions in absolute mass were observed across all fat depots in the BDNF‐treated mice. BDNF treatment decreased adiposity: fat mass, relative to body weight, by 68% for iWAT, 79% for rWAT and gWAT (Figure 4a). Although the absolute weight of liver was lower in BDNF group, the relative liver mass when normalized to body weight was significantly increased compared to YFP mice (Figure 4b). The lean phenotype of BDNF‐treated mice was associated with a serum biomarker profile featured as sharp drop of leptin and insulin, and rise of adiponectin and IGF‐1 (Figure 4c).

### 2.5 Brain gene expression

Sustained hypothalamic BDNF overexpression was confirmed by quantitative RT–PCR at the end of the study (Figure 4d). Further expression profiling of the whole hypothalamus revealed significant upregulation of insulin receptor (Insr) and Mc4r (Figure 4e). Interestingly, inflammation‐modulatory genes were collectively downregulated in BDNF overexpressing hypothalamus including Il1b (encoding interleukin‐1β), Il6 (encoding interleukin‐6), Il2rg (encoding interleukin‐2 receptor γ), Ly6d (encoding lymphocyte antigen 6 family member D), Cxcl10 (encoding C‐X‐C motif chemokine 10), and Nfkbia (encoding NFκB inhibitor α; Figure 4e). The gene expression signature observed in the hypothalamus, namely upregulation of insulin receptor and downregulation of inflammation‐modulatory genes was not observed in amygdala (Figure 4f) or hippocampus (Figure 4g).

### 2.6 Adipose remodeling

**Figure 5:**
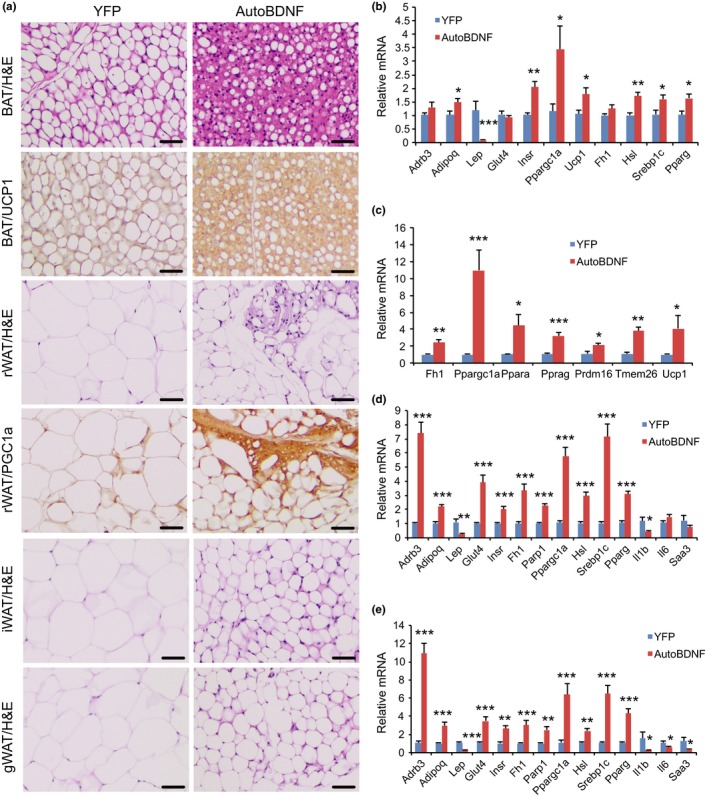
Hypothalamic gene transfer of BDNF remodels adipose tissues. (a) Representative H&E staining and immunohistochemistry of UCP1 and PGC‐1α. Scale bar, 50 µm. (b) Gene expression profiling of BAT. (c) Gene expression profiling of rWAT. (d) Gene expression profiling of iWAT adipocytes. (e) Gene expression profiling of gWAT adipocytes. *n* = 5 per group. Error bars represent mean ± *SEM*. *p < 0.05. **p < 0.01. ***p < 0.001

**Figure 6:**
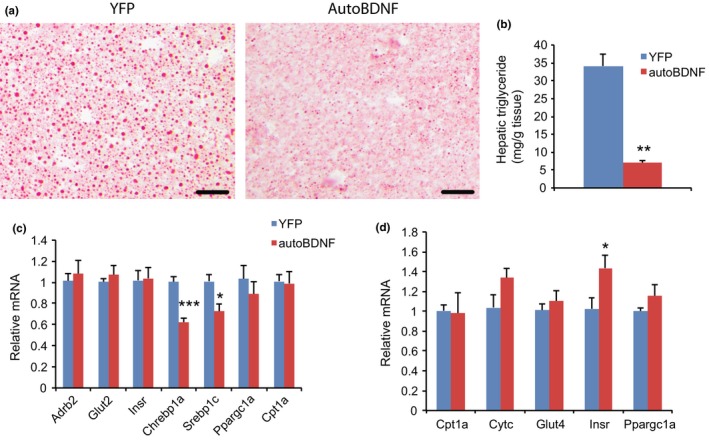
Hypothalamic gene transfer of BDNF reduces hepatic steatosis. (a) Representative oil red O staining. Scale bar = 50 µm. (b) Hepatic triglyceride levels. *n* = 8 for YFP, 9 for autoBDNF. (c) Gene expression profiling of liver. *n* = 5 per group. (d) Gene expression profiling of skeletal muscle. *n* = 5 per group. Error bars represent mean ± *SEM*. *p < 0.05. **p < 0.01. ***p < 0.001.

Aging is associated with a decline in BAT activity (Enerback, 2010). The BATs of 19 months old YFP mice appeared pale whereas the BAT in BDNF mice was darker. H&E staining revealed the BAT of BDNF mice maintained typical BAT morphology of younger mice and was devoid of white adipocyte infiltration often associated with aging (Figure 5a). The morphological changes of BAT were associated with robust regulation of BAT gene expression (Figure 5b). Leptin expression was reduced by over 90% while adiponectin expression was upregulated. Insulin receptor expression was also significantly upregulated whereas glucose transporter type 4 (Glut4), the major type of glucose transporter in adipose tissue, was not different between the two groups (Figure 5b). Both the lipolytic gene Hsl (encoding hormone‐sensitive lipase) and the lipogenic gene Srebp1c (encoding sterol regulatory element‐binding protein 1c) were upregulated in BDNF mice. BAT dissipates energy via releasing chemical energy from mitochondria in the form of heat. This process is primarily mediated by uncoupling protein‐1 (UCP1) that is a specific BAT marker (Enerback et al., 1997). UCP1 was significantly upregulated by BDNF treatment suggesting the preservation of proper BAT functions against aging‐related loss (Figure 5a,b, Supporting Information Figure S1). The transcriptional coactivator peroxisome proliferator‐activated receptor γ coactivator 1‐α (PGC‐1α) switches cells from energy storage to energy expenditure by inducing mitochondrial biogenesis and genes involved in thermogenesis (Puigserver et al., 1998). Ppargc1a (encoding PGC‐1α) was increased over threefold in the BAT of BDNF mice (Figure 5b). BDNF treatment similarly induced Ppargc1a expression in all three WAT depots examined (Figure 5c–e), but not in the liver (Figure 6c) or muscle (Figure 6d). In the rWAT of BDNF mice, clusters of beige cells were observed by H&E staining (Figure 5a). Ppargc1a mRNA level was upregulated approximately 10‐fold in rWAT of BDNF mice (Figure 5c) and the increased PGC‐1α protein level was confirmed by immunohistochemistry (Figure 5a, Supporting Information Figure S1). Additional beige gene markers Prdm16 (encoding PR domain zinc finger protein 16) and Tmem26 (encoding transmembrane protein 26) were also upregulated in rWAT of BDNF mice (Figure 5c). Major regulators of lipid metabolism, Ppara (encoding peroxisome proliferator‐activated receptor α) and Pparg (encoding peroxisome proliferator‐activated receptor γ), were also upregulated in BDNF animals. We isolated adipocytes from subcutaneous iWAT and visceral gWAT depots for gene expression profiling. The gene expression signatures induced by hypothalamic BDNF gene transfer were similar between iWAT (Figure 5d) and gWAT adipocytes (Figure 5e), including upregulation of Adrb3 (encoding β3 adrenergic receptor), Adipoq (encoding adiponectin), Glut4 (encoding glucose transporter 4), Insr, Hsl (encoding hormone‐sensitive lipase), Srebp1c and Pparg. Fh1 (encoding mitochondrial fumarate hydratase) and Parp1 (encoding poly ADP‐ribose polymerase 1) are associated with caloric restriction‐induced metabolic adaption (Mitchell et al., 2016). BDNF treatment induced both Fh1 and Parp1 in iWAT and gWAT adipocytes (Figure 5d,e). Leptin expression was decreased by 70% in both depots. Inflammation‐modulatory genes Il1b, Il6 and Saa3 (encoding serum amyloid 3A) were highly suppressed in gWAT adipocyte (Figure 5e). Histology and quantification showed that the size of white adipocyte in BDNF mice was much smaller than that in YFP mice (Figure 5a, Supporting Information Figure S2).

### 2.7 BDNF treatment inhibits liver steatosis

Aging is associated with hepatic steatosis (Sheedfar, Biase, Koonen, & Vinciguerra, 2013). Oil red O staining revealed lipid accumulation in the livers of YFP mice of 19 months of age. In contrast, BDNF treatment diminished liver steatosis (Figure 6a). Lipid extraction and quantification revealed that hepatic triglyceride levels were reduced by approximately 80% in BDNF mice compared to YFP mice (Figure 6b). The major transcription factors that regulate de novo lipogenesis enzymes, Chrebp1a (encoding carbohydrate‐responsive element‐binding protein 1a) and Srebp1c, were significantly downregulated in the liver of BDNF mice consistent with reduced steatosis (Horton, Goldstein, & Brown, 2002; Iizuka, Bruick, Liang, Horton, & Uyeda, 2004; Figure 6c). Limited changes were found in skeletal muscle (Figure 6d).

## 3 DISCUSSION

Our data demonstrate that hypothalamic gene transfer of BDNF prevents aging‐associated metabolic decline including weight gain, loss of BAT function, hepatic steatosis, and impaired glucose tolerance. The physiological autoregulatory BDNF expression vector achieved a sustainable plateau of weight loss and leanness in aged mice maintained on a normal diet. Food intake was decreased proportionally to body weight in BDNF‐treated mice suggesting that hypophagia is unlikely to be the primary cause of weight loss. We previously reported that hypothalamic BDNF overexpression increased physical activity, measured by CLAMS, in diet‐induced obesity (DIO) mice (Cao et al., 2009) while having no significant effect in MC4R deficient mice (Siu et al., 2017). BDNF treatment substantially reduced body weight and adiposity in both models. In this study, hypothalamic BDNF overexpression significantly decreased physical activity whereas oxygen consumption was increased in aged mice of normal weight. These findings suggest that some features of hypothalamic BDNF in the regulation of energy balance depend on the age, diet, and adiposity status. Identifying the underlying mechanisms warrants further investigations. Nevertheless, increased oxygen consumption concurrent with decreased physical activity in CLAMS indicates that BDNF treatment elevates basal metabolic rate in the aged mice maintained on normal diet.

Cai and colleagues propose a conceptual model in which hypothalamic microinflammation is a common basis of metabolic syndrome and aging (Tang, Purkayastha, & Cai, 2015). The hypothalamus orchestrates actions of neural pathways and neuroendocrine hormones that regulate energy balance and nutrient homeostasis. Chronic overnutrition induces inflammation‐like changes in the hypothalamus mediated by low‐degree activation of proinflammatory NFκB and its upstream IKKβ (Kleinridders et al., 2009; Purkayastha, Zhang, & Cai, 2011; Thaler et al., 2012; Zhang et al., 2008). The atypical neural inflammatory changes interrupt the central regulation of energy balance, glucose homeostasis and promote the core features of metabolic syndromes. Notably, low‐grade inflammation is also a hallmark of aging, and systemic inflammation is negatively correlated with human longevity (Harris et al., 1999; Khabour & Barnawi, 2010). Recent studies have demonstrated that hypothalamic microinflammation promotes systemic aging (Tang & Cai, 2013; Zhang et al., 2013). Thus, NFκB‐dependent hypothalamic microinflammation is proposed to represent a shared means through which conditions of dietary excess and aging can mediate the consequent development of metabolic and aging‐related diseases (Tang et al., 2015). Our previous data in DIO mice and the new finding of this study in aged mice of normal weight demonstrate the suppression of NFκB pathway genes by hypothalamic BDNF treatment. Whether this effect contributes to the systemic metabolic modulations requires further investigation. Furthermore, the collective downregulation of the inflammation‐modulatory genes was observed specifically in the hypothalamus but not in the amygdala or hippocampus of the same mouse, suggesting the suppression is likely downstream of BDNF signaling instead of feedback from systemic metabolic improvement. AAV1 vector predominantly transduces neurons (Wang, Wang, Clark, & Sferra, 2003) and therefore BDNF was primarily overexpressed in neurons in this study. However, the current study is unable to distinguish between the autocrine and paracrine effects of BDNF since BDNF can be secreted from transduced neurons. We are currently using AAV1 to deliver a dominant‐negative TrkB receptor (Cao et al., 2011) to the hypothalamus of aged mice. The specific blockade of BDNF signaling in the hypothalamic neurons will reveal the role of neuronal BDNF signaling in hypothalamic microinflammation and systemic aging.

Both BAT and WAT showed robust remodeling in BDNF‐treated mice. Shared features across all the adipose depots included suppression of leptin, stimulation of adiponectin, upregulation of insulin receptor and mitochondrial genes. Interestingly, gene expression profiling showed upregulation of lipolytic and lipogenic genes as well as genes involved in lipid flux and fatty acid oxidation in BDNF‐treated mice. In addition, BDNF treatment suppressed inflammation‐modulatory genes in adipocytes isolated from both subcutaneous and abdominal adipose depots. It will be interesting to investigate whether hypothalamic BDNF modulates adipose senescence and functional decline associated with aging.

One new finding of this study is the improved glucose tolerance by BDNF treatment in aged mice on normal diet. Our previous studies show improvement in glycemic control in obesity and diabetes models but not in young mice of normal weight (data not shown). This improved glycemic control was associated with decreased adiposity, improved adipokine profile (lower leptin, higher adiponectin), and alleviation of hepatic steatosis in aged mice. The adipose remodeling may contribute to the improved glucose tolerance directly by elevation of glucose uptake to the adipose tissues or indirectly by crosstalk to the liver. Of note, the drastic shrinkage of fat and hypoleptinemia in BDNF‐treated mice were not associated with lipodystrophy. On the contrary, the hepatic triglyceride level was decreased by fivefold, and the transcription factors critical for de novo lipogenesis were significantly downregulated in the livers of BDNF‐treated mice. In a rodent model of uncontrolled insulin‐deficient diabetes, Meek and colleagues report that infusion of BDNF to either the lateral cerebral ventricle or the VMH attenuates diabetic hyperglycemia via an insulin‐independent inhibition of hepatic glucose production (Meek et al., 2013). Future studies will elucidate whether a hypothalamic BDNF‐liver axis directly modulates liver glucose and lipid metabolism and thereby contributes to glycemic control in aged animals.

With respect to the effects on behavior, most studies associate a reduction in BDNF with cognitive deficits. Postnatal knockout of *Bdnf* leads to increased anxiety along with obesity (Rios et al., 2001), while forebrain‐specific deletion results in impaired spatial learning and certain discrimination tasks (Gorski, Balogh, Wehner, & Jones, 2003). Moreover, low serum levels of BDNF are correlated with depression in human patients (Karege et al., 2005; Shimizu et al., 2003). However, scarce evidence is available regarding the role of hypothalamic BDNF in emotionality. To our knowledge, this study is the first assessing hypothalamic BDNF‐induced behavioral adaptations in aging. We examined a battery of anxiety and depression behavior tests and showed that hypothalamic BDNF treatment significantly reduced anxiety‐ and depression‐like behaviors. Transgene was expressed mainly in the ARC and VMH nuclei of the hypothalamus in this study, which might increase the BDNF protein level in the adjacent dorsomedial hypothalamus (DMH). The DMH is a brain area not only involved in physiological functions such as metabolism and environmental threats but is also critically involved in behavioral regulation, particularly fear, anxiety, and panic‐like disorders (Shekhar, Sims, & Bowsher, 1993; Silva et al., 2014). Recently, it was reported that loss of corticotropin‐releasing hormone (Crh) in the paraventricular hypothalamus (PVH) results in reduced anxiety behaviors (Zhang et al., 2016). Future research is required to elucidate the role of BDNF in these specific hypothalamic nuclei regarding anxiety. Alternatively, we have reported that hypothalamic BDNF modulates the hypothalamic‐pituitary‐adrenal (HPA) axis partially mediating the EE's regulation of T cell immunity (Xiao et al., 2016). It will be interesting to investigate whether hypothalamic BDNF's modulation of the HPA axis contributes to the anti‐depression effect in the aged animals whose stress response system becomes less agile and dysfunctional. Although no change in BDNF expression was found in either the hippocampus or the amygdala, it is still possible that global improvement of metabolism induced by hypothalamic BDNF overexpression indirectly influences other limbic structures, including the prefrontal cortex, hippocampus, nucleus accumbens, ventral striatum, amygdala, and hypothalamus (Russo, Murrough, Han, Charney, & Nestler, 2012) and thereby affects brain functions and behaviors (de Noronha et al., 2016).

In conclusion, hypothalamic BDNF gene transfer with an autoregulatory AAV vector prevents aging‐related weight gain, reduces adiposity, increases energy expenditure, improves glycemic control, alleviates liver steatosis, suppresses inflammatory genes in the hypothalamus and adipose tissues, and decreases anxiety‐ and depression‐like behaviors. This long‐term study provides efficacy and safety evidence targeting hypothalamic BDNF for healthy aging.

## 4 EXPERIMENTAL PROCEDURES

### 4.1 Animals

National Institute on Aging, Aged Rodent Colonies, provided female C57Bl/6 mice, 12 months of age. Mouse litters were group housed (no more than five per cage) in a 12:12 light:dark cycle with ad libitum access to standard rodent chow and water in a humidity‐ and temperature‐controlled environment. All animal experiments were carried out in compliance and conform to the regulatory standards of the Ohio State University Institutional Animal Care and Use Committee.

### 4.2 rAAV vector constructs

The recombinant AAV (rAAV) vector plasmid contains the following expression cassette flanked by inverted terminal repeats (ITRs): cytomegalovirus enhancer plus chicken ß‐actin promoter (CBA), woodchuck posttranscriptional regulatory element (WPRE), and bovine growth hormone polyadenosine (BGH polyA) tail. Between the CBA and polyA is a multiple cloning site (MCS) in which HA‐tagged human *Bdnf* (AAV‐HA‐BDNF, referred to simply as BDNF) or destabilized yellow fluorescent protein control (AAV‐dsYFP, referred to as YFP) is inserted. The second cassette with human AGRP minimal promoter driving miR‐*Bdnf* expression is cloned after the first cassette containing BDNF within the ITRs and referred to as autoBDNF. All vectors were packaged into serotype 1 capsids and purified by iodixanol gradient centrifugation.

### 4.3 Stereotaxic surgery

The 10 or 12‐month‐old female C57Bl/6 mice were randomly assigned to receive AAV‐autoBDNF or AAV‐YFP. Mice were anaesthetized with a single intraperitoneal dose of ketamine/xylazine (100 mg/kg and 20 mg/kg) and secured via ear bars and incisor bar on a Kopf stereotaxic frame (Tujunga, CA). A single midline incision was made through the scalp to expose the skull and two small holes were made with a dental drill above the injection sites. rAAV vectors were adjusted to 1.0 × 10^10^ genomic particles per microliter and then administered bilaterally by a 10 µl Hamilton syringe (Reno, NV) attached to a Micro4 Micro Syringe Pump Controller (World Precision Instruments, Sarasota, FL) at a rate of 100 nl/min for a total of 0.5 µl into the arcuate/ventromedial hypothalamus (AP: −1.20 mm, ML: ±0.50 mm, DV: −6.20). When the infusion was finished, the syringe was slowly retracted from the brain, the scalp was sutured, and mice were administered buprenorphine for pain relief (0.05 mg/kg). Animals were returned to clean cages with hydrogel (ClearH_2_O, Westbrook, ME) provided for supplemental hydration resting atop a 37°C heating pad and carefully monitored postsurgery until fully recovered.

### 4.4 Food intake and body weight

Following surgeries, body weight and food intake‐ on normal chow diet‐ were recorded every 5–7 days. Animals injected with the same vector remained housed together postsurgery. Food intake was averaged per mouse per week in each cage. Mice were monitored up to 63 days postsurgery for short‐term study while 194 days post injection for long‐term study.

### 4.5 Body temperature

Rectal temperature was measured at 2 p.m. for all mice after 5 min of sedation with 2.5% isoflurane. The Physitemp BAT −12 rectal thermometer (Clifton, NJ) remained in place for 30 s to allow temperature to stabilize before being recorded. Mice were then returned to their home cages to recover.

### 4.6 Glucose tolerance test

Glucose tolerance test was performed after an overnight fast. Mice were injected intraperitoneally with glucose solution (2 mg/kg body weight). Tail blood was collected at 0, 15, 30, 60, 90, and 120 min after glucose injection. Blood glucose concentrations were measured with a portable glucometer (Bayer Contour Next, Parsippany, NJ).

### 4.7 Energy expenditure

At 20 weeks post‐AAV injection, mice underwent indirect calorimetry using the Oxymax Comprehensive Lab Animal Monitoring System (CLAMS, Columbus Instruments, Columbus, OH). Mice, singly housed with access to ample food and H_2_O, acclimated to the metabolic chambers for 1 day and then behavioral and physiological parameters (O_2_ consumption, CO_2_ production, respiratory exchange ratio, and physical activity) were recorded at room temperature for up to 2 days.

### 4.8 Novelty‐suppressed feeding

Mice were fasted overnight with food removed at 17:30 hr. The testing phase was conducted the next day at 14:00 hr. Mice were individually placed into a brightly lit novel open cage (38 cm × 24 cm × 22 cm). A round piece of white filter paper (7 cm diameter) was placed in the center of the cage with a single preweighed food pellet. The latency to consumption (first bite of thecenter of the cage with food pellet) was recorded as a measure of anxiety‐like behavior. The cutoff time was 10 min. To assess if there was any difference in consumptive drive, each mouse was placed in a standard cage with the preweighed food pellet after its first bite or at cutoff time if it failed to eat within 10 min. The amount of food consumed in 5 min was measured.

### 4.9 Cold‐induced defecation

A large container was filled halfway with ice. Small novel cages (28 × 16 × 12 cm^3^) were placed on top of the ice. Mice were placed individually into the cages, and then lids were placed on top. After 20 min, mice were removed and the number of fecal matter was counted as a measure of anxiety‐like behavior. Mice were allowed to recover in a cage partially on a heating pad for 1 hr prior to returning to its home cage. All cages used were cleaned with 70% ethanol between trials.

### 4.10 Tail suspension test

A small cylindrical tube (Becton, Dickinson and Company, Franklin Lakes, NJ) was slipped over the mouse tail to prevent climbing motion and escape from the test. Mice were suspended in air individually by tape attached to a shelf (64 cm height) for 6 min. Trials were video‐recorded and a blinded experimenter scored the amount of time mice remained immobile as a measure of depressive‐like behavior.

### 4.11 Forced swim test

Mice were placed individually in a transparent cylinder (21 cm diameter, 24 cm height) containing water (25 ± 2°C) to a depth of 15 cm for 6 min. At the end of each trial, mice were dried and returned to their home cage. Trials were video‐recorded and a blinded experimenter scored the amount of time mice remained immobile as a measure of depressive‐like behavior.

### 4.12 Serum harvest and analysis

Truncal blood was collected at 10 a.m. following decapitation at sacrifice. Serum was allowed to clot on ice for at least 30 min before centrifugation at 94 *g* (Eppendorf Centrifuge 5424R) for 20 min at 4°C. Serum was collected and stored at −20°C until further analysis. Biomarkers were analyzed with the following kits: glucose and triglyceride with Cayman Colorimetric Assay kits (Ann Arbor, MI), leptin, IGF‐1, and adiponectin/Acrp30 with R&D DuoSet ELISA Development Systems (Minneapolis, MN), insulin with Alpco Mouse Ultrasensitive Insulin ELISA (Salem, NH), growth hormone with Alpco mouse/rat growth hormone ELISA (Salem, NH).

### 4.13 Adipose tissue histology and immunohistochemistry

Adipose tissue depots were fixed in 10% formalin (w/v), then transferred to 70% ethanol, embedded in paraffin, and sectioned at 4 µm thickness. Hematoxylin and eosin (H&E) staining was then performed, and stained sections were imaged on a Zeiss Axioskop 40 light microscope (Goettingen, Germany) to assess fat cell morphology and size. Paraffin‐embedded sections were subjected to citrate‐based antigen retrieval followed by incubations with antibodies against UCP1 (Abcam ab10983, 1:1,000), or PGC‐1α (Abcam ab54481, 1:250). The sections were visualized with DAB and counterstained with hematoxylin.

### 4.14 Liver histology

Liver was dissected at sacrifice, snap frozen on dry ice, and stored at −80°C. For sectioning, liver tissue was embedded in O.C.T. (Sakura Finetek, Torrance, CA) before being sectioned into 15 µm slices on a Leica cryostat. Lipids in frozen liver sections were then stained with an Oil Red O solution (Sigma, St. Louis, MO).

### 4.15 Perfusion

At 63 days post‐AAV injection, mice were intracardially perfused with 4% paraformaldehyde (PFA, Sigma, St. Louis, MO) in PBS, and fixed brains were incubated in 4% PFA on a rocker overnight at 4°C. The next day, brains were rinsed in PBS three times before being submerged in 30% sucrose in PBS and 0.03% sodium azide for at least 3 days on a rocker at 4°C. Brain tissues were then embedded in O.C.T. (Sakura Finetek, Torrance, CA) before being sectioned into 30 µm slices on a Leica cryostat. Fluorescence microscopy was performed on a Zeiss microscope (Thornwood, NY), and images were captured with Zen Pro software.

### 4.16 Isolation of adipocytes

Based on previously described methods (Sackmann‐Sala, Berryman, Munn, Lubbers, & Kopchick, 2012), adipose tissues were dissected and transferred to 12 well culture plate containing Krebs‐Ringer HEPES buffer (5 mM d‐glucose, 2% BSA, 135 mM NaCl, 2.2 mM CaCl2, 1.25 mM MgSO4, 0.45 mM KH2PO4, 2.17 mM Na2HPO4, and 10 mM HEPES (pH 7.4)), then minced to a fine consistency. Collagenase II (Sigma) at 1.2 mg/ml was added and the fat pads mixture was incubated at 37°C with shaking at 150 rpm up to 30 min. The mixture was spun at 800 rpm for 5 min after passing through strainer (100 µm mesh size). The mature adipocytes floating at the top were collected and washed with above‐mentioned buffer twice. The adipocytes were stored at −80°C until further analysis.

### 4.17 Quantitative RT–PCR

Hypothalamus, amygdala, and hippocampus were block dissected from mouse brains at sacrifice. Tissue was sonicated, and RNA was isolated using the Qiagen RNeasy Mini kit with RNase‐free DNase treatment (Germantown, MD). Next, cDNA was reverse transcribed using TaqMan Reverse Transcription Reagents (Applied Biosystems, Foster City, CA). Finally, quantitative PCR was carried out on StepOnePlus Real‐Time PCR System (Applied Biosystems) with the Power SYBR Green PCR Master Mix (Applied Biosystems). We calibrated data to endogenous control Actb for adipose tissues and adipocyte, Ppia for liver, Gapdh for muscle, Hprt1 for hypothalamus and hippocampus, Ppia for amygdala, and quantified the relative gene expression using the 2^−ΔΔCT^ method. Primer sequences are available on request.

### 4.18 Hepatic triglyceride measurement

Lipid was extracted from liver by chloroform/methanol (2:1 v/v), followed by rinse in 50 mM NaCl and CaCl (0.36 M)/Methanol (1:1 v/v; Folch, Lees, & Sloane Stanley, 1957). Hepatic triglycerides quantification was carried out using a WAKO Diagnostics kit (Mountain View, CA).

### 4.19 BDNF ELISA

Hypothalamic block dissections were homogenized in ice‐cold Pierce RIPA buffer containing Calbiochem protease inhibitor cocktail III (San Diego, CA). The homogenates were centrifuged and the protein content was measured using BCA kit (Pierce Biotechnology, Rockford, IL). BDNF protein levels were measured using R&D DuoSet ELISA Development Systems (Minneapolis, MN).

### 4.20 Statistical analysis

Data are expressed as mean ± *SEM*. We used Prism Mac version 6.0f software (GraphPad, La Jolla, CA) and SPSS Statistics v24.0.0.0 (IBM, Armonk, NY) to analyze the following: student's *t* test for body weight or food intake at single time points, adiposity, body temperature, organ weights, serum ELISAs, behavior, and quantitative RT–PCR data. Mixed analysis of variance was performed on time course measurements (body weight, VO_2_, RER, physical activity, GTT).

## **AUTHOR** **CONTRIBUTIONS**

T.M, W.H., X.L., J.J.S., N.J.Q, and R.X.: carried out the research and interpreted the results. L.C.: conceived the concept, designed the studies, interpreted the results, and wrote the manuscript. All authors approved the manuscript.
