# Supplementary figures and images for: Hypothalamic gene transfer of BDNF promotes healthy aging in mice
Source: Aging Cell. 2018 Dec 26;18(2):e12846. doi: 10.1111/acel.12846 (PMC6413658; doi:10.1111/acel.12846)

Fig S1

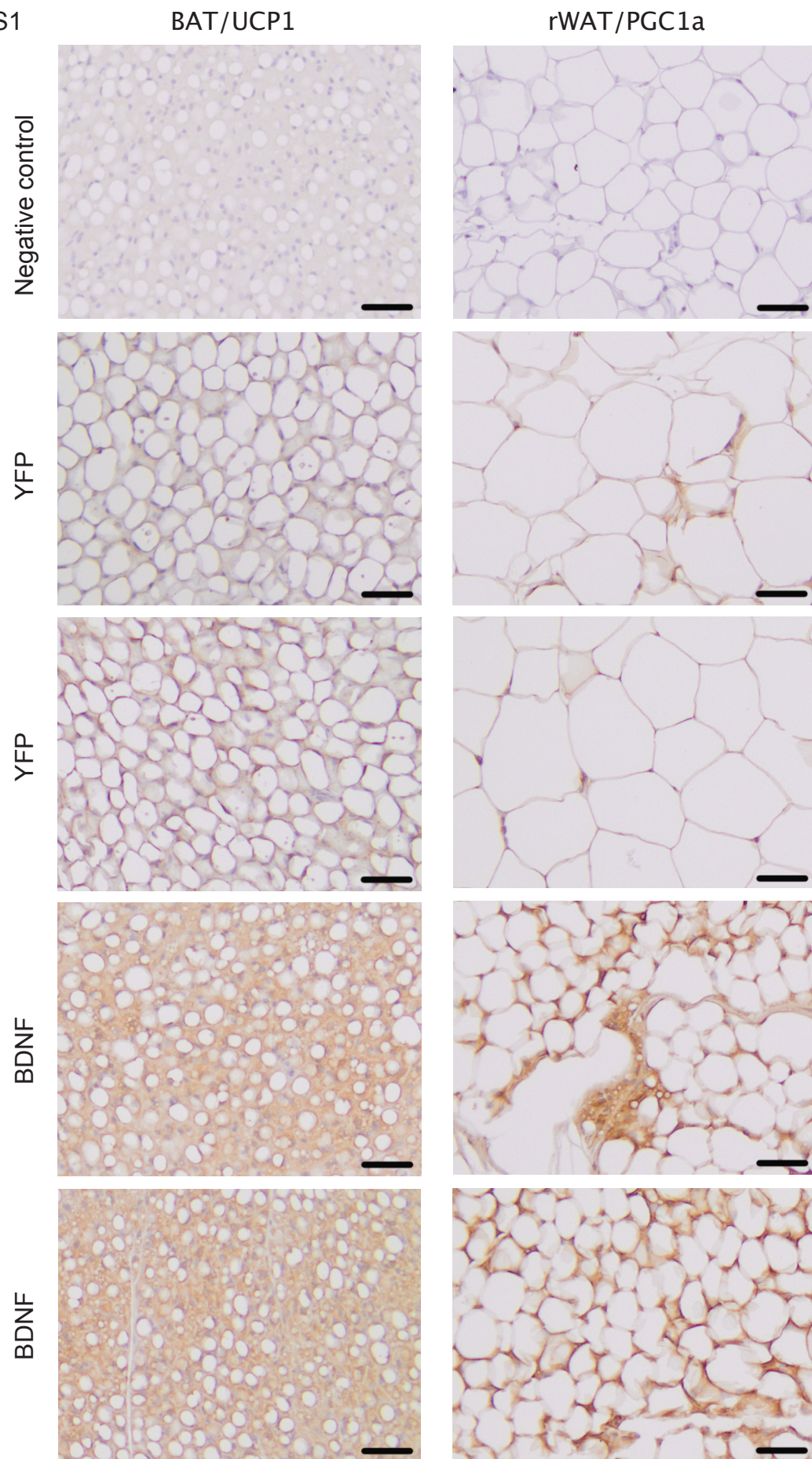

Supplement: Supplementary file 1 [file ACEL-18-e12846-s001.pdf]

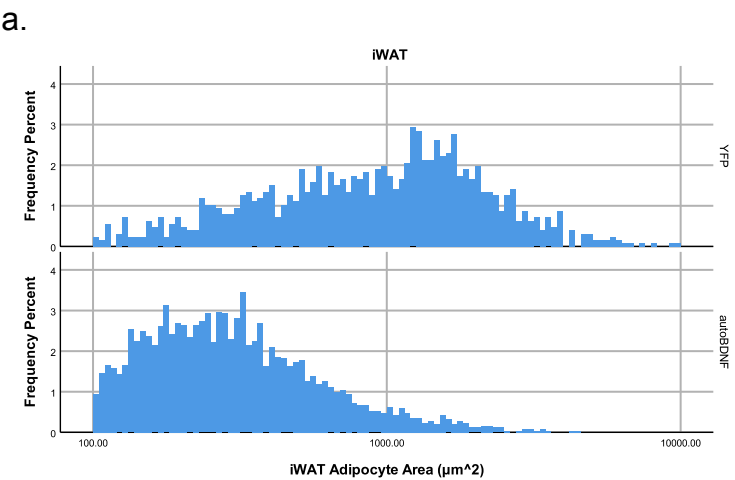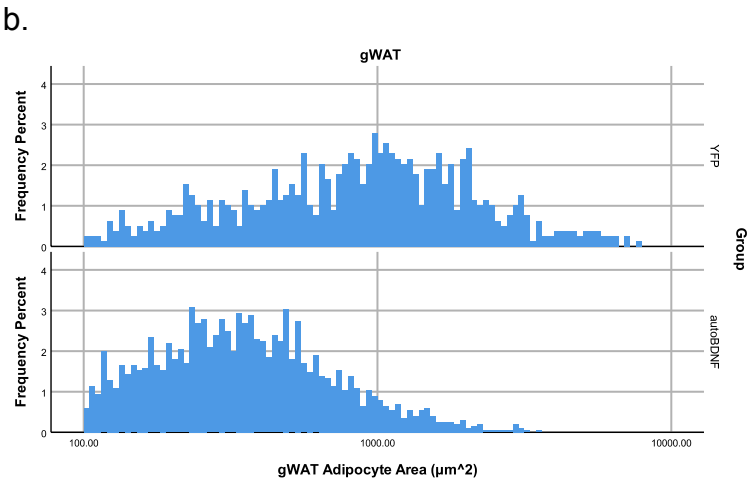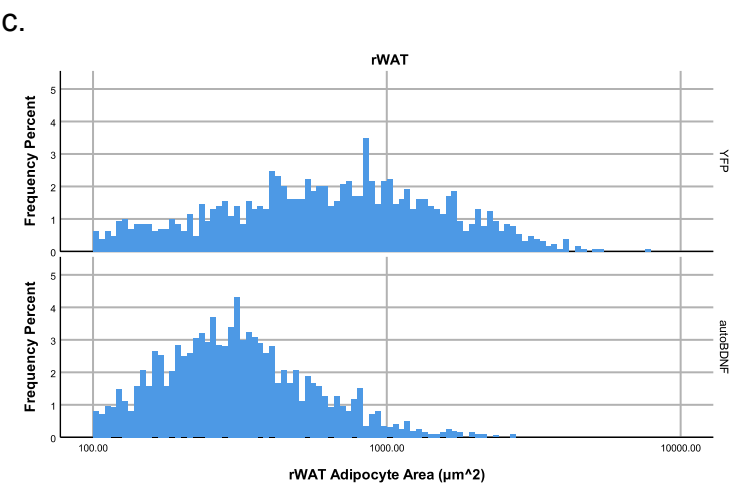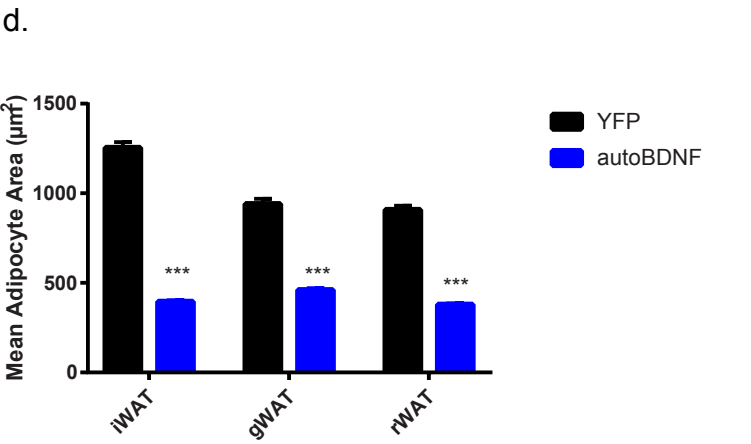

Supplement: Supplementary file 2 [file ACEL-18-e12846-s002.pdf]
