# Supplementary material for: Hypothalamic gene transfer of BDNF promotes healthy aging in mice
Source: Aging Cell. 2018 Dec 26;18(2):e12846. doi: 10.1111/acel.12846 (PMC6413658; doi:10.1111/acel.12846)
Supplement: Supplementary file 3 [file ACEL-18-e12846-s003.docx]

Supplemental Information

“Hypothalamic gene transfer of BDNF promotes healthy aging in mice”

Travis McMurphy, Wei Huang, Xianglan Liu, Jason J. Siu, Nicholas J. Queen, Run Xiao, and Lei Cao

**Experimental Procedures**

**H&E staining and image analysis**. Portion of fat depots were fixed in 10% formalin. Paraffin sections (4 μm) were processed and H&E staining were performed by histology core of the Ohio State University Comprehensive Cancer Center. Adipose tissue sections were imaged at 10x magnification using a Nikon Eclipse 50i microscope with an Axiocam 506 color camera attachment through ZEN 2 software. For each field, adipocyte area was measured in FIJI using a semi-automated custom method based on the Adiposoft algorithm (Galarraga et al., 2012; Rueden et al., 2017; Schindelin et al., 2012). Briefly, each image was split into H&E color channels by Colour Deconvolution, then made binary by the Otsu thresholding algorithm (Otsu, 1979). The images were then filtered with opening and median operators with the Morphological Filters plugin to sharpen cell boundaries (Legland et al., 2016). Automated particle subtraction and manual removal of extraneous particles were performed to reduce noise. In white adipose tissue, a cutoff of 100µm^2^ was used as the minimum size for an adipocyte.

**Figure legend**

**Supplemental Figure 1.** Immunohistochemistry of UCP1 and PGC-1α in adipose tissues. Representative images from two mice of each group. Scale bar, 50 μm.

**Supplemental Figure 2.** Quantification of cell size in iWAT (a), gWAT (b), and rWAT (c). (d) Average size of adipocytes. n=4-6 images per fat pad per mouse, n=3 mice per group, total cell numbers analyzed per fat pad per group 1263~2527. Data are means ± SEM. *** P<0.001.

Galarraga, M., Campion, J., Munoz-Barrutia, A., Boque, N., Moreno, H., Martinez, J.A., Milagro, F., and Ortiz-de-Solorzano, C. (2012). Adiposoft: automated software for the analysis of white adipose tissue cellularity in histological sections. J Lipid Res *53*, 2791-2796.

Legland, D., Arganda-Carreras, I., and Andrey, P. (2016). MorphoLibJ: integrated library and plugins for mathematical morphology with ImageJ. Bioinformatics *32*, 3532-3534.

Otsu, n. (1979). A Threshold Selection Method from Gray-Level Histograms. IEEE Trans Syst Man Cybern *9*, 62-69.

Rueden, C.T., Schindelin, J., Hiner, M.C., DeZonia, B.E., Walter, A.E., Arena, E.T., and Eliceiri, K.W. (2017). ImageJ2: ImageJ for the next generation of scientific image data. BMC Bioinformatics *18*, 529.

Schindelin, J., Arganda-Carreras, I., Frise, E., Kaynig, V., Longair, M., Pietzsch, T., Preibisch, S., Rueden, C., Saalfeld, S., Schmid, B.*, et al.* (2012). Fiji: an open-source platform for biological-image analysis. Nat Methods *9*, 676-682.
